# Insights into *In Vitro* Wound Closure on Two Biopolyesters—Polylactide and Polyhydroxyoctanoate

**DOI:** 10.3390/ma13122793

**Published:** 2020-06-20

**Authors:** Tomasz Witko, Daria Solarz, Karolina Feliksiak, Katarzyna Haraźna, Zenon Rajfur, Maciej Guzik

**Affiliations:** 1 Faculty of Physics, Astronomy and Applied Computer Science, Jagiellonian University, Lojasiewicza 11, 30-348 Krakow, Poland; tomasz.witko@ikifp.edu.pl (T.W.); daria.solarz@doctoral.uj.edu.pl (D.S.); karolina.feliksiak89@gmail.com (K.F.); 2 Jerzy Haber Institute of Catalysis and Surface Chemistry Polish Academy of Sciences, Niezapominajek 8, 30-239 Krakow, Poland; ncharazn@cyf-kr.edu.pl

**Keywords:** wound healing, actin, migration, polyhydroxyalkanoates, polyhdroxyoctanoate, polylactide

## Abstract

Two bio-based polymers have been compared in this study, namely: polylactide (PLA) and polyhydroxyoctanoate (PHO). Due to their properties such as biocompatibility, and biointegrity they are considered to be valuable materials for medical purposes, i.e., creating scaffolds or wound dressings. Presented biopolymers were investigated for their impact on cellular migration strategies of mouse embryonic fibroblasts (MEF) 3T3 cell line. Advanced microscopic techniques, including confocal microscopy and immunofluorescent protocols, enabled the thorough analysis of the cell shape and migration. Application of wound healing assay combined with dedicated software allowed us to perform quantitative analysis of wound closure dynamics. The outcome of the experiments demonstrated that the wound closure dynamics for PLA differs from PHO. Single fibroblasts grown on PLA moved 1.5-fold faster, than those migrating on the PHO surface. However, when a layer of cells was considered, the wound closure was by 4.1 h faster for PHO material. The accomplished work confirms the potential of PLA and PHO as excellent candidates for medical applications, due to their properties that propagate cell migration, vitality, and proliferation—essential cell processes in the healing of damaged tissues.

## 1. Introduction

The investigation of biopolymers has been reserved for biochemists and molecular biologists for over half of a century. Nevertheless, during the last decade, the soft matter physics, biophysics, chemists, and material scientists have been seized to this research field [1]. The current interest in developing novel bio-based materials has motivated an increasing need for biological and medical studies for a variety of clinical applications [2]. One of the intensely studied and widely used group of biopolymers are polyesters derived from nature. Firstly, these are polyhydroxyalkanoates (PHAs) of bacterial origin [3], a class of optically active biodegradable polyesters [4]. They are stored inside the microbes’ cells in the form of granules, as internal carbon and energy storage compounds and part of their survival mechanism [5], which are easy to recover and purify [6]. Another example of nature-derived polyester is polylactide (PLA). These polymers are synthetized from lactic acid monomers produced by microorganisms. PLA is one of the most innovative materials being actively investigated for a wide range of industrial applications. This biodegradable and biocompatible polymer is a linear aliphatic thermoplastic polyester. PLAs are synthesized and obtained from renewable agricultural resources [7] and are an attractive choice for applications involving human interface because of their biocompatibility and biodegradability [8]. There are many commercialized PLA products in today’s market; their variety and consumption are increasing rapidly. 

Due to its properties PLA has attracted attention and interest as novel material for a wide range of applications also in biomedical and pharmaceutical applications, e.g., drug delivery systems, surgical sutures, implants for bone fixation, etc. [9]. They are widely used in orthopedic medicine, soft tissue repair, synthetic grafts, etc. PLA used as a bio-absorbable surgical device was reported by Kulkarni, et al. in 1971 [10]. Orthopedic implants made of PLA-calcium-phosphate-glass-fiber composites degrade faster as pure PLA or glass fibers [11]. The combination of PLA and polyhydroxyoctanoate (PHO) with hydroxyapatite (HA) ceramic and aluminum-calcium-phosphorous oxide ceramic significantly enhances its mechanical properties and can find application in repairing and replacement of soft and hard tissues [9,12]. Both PLA and PHO can be used to produce bone scaffolds [13] and thanks to their “smart” properties i.e., piezoelectric characteristics of PHO or PLA can act as electro stimulating device without the need for an external power source or any cables [14].

An ideal wound patch should have a suitable strength as well as flexibility and hardness, enabling imitation of the natural tissue environment, during processes of skin regeneration [15]. Young’s modulus values of 6.2–7.4 GPa of PLA indicate that it is a non-elastomeric, rigid material. PLA hardness values in the range from 7 to 11 MPa implies that it is also brittle. Therefore, the PLA-based patches (i.e., solid thin films) would be problematic in applying it to the wound site, which is a necessary parameter in order to avoid infection or inflammation [16]. Nevertheless, this material, in combination with other polymers, was shown to be suitable for construction of dressing materials [17,18,19,20]. PHO Young’s modulus values of 33–41 MPa [21] indicate that it can mimic the mechanical properties of selected tissues and organs inside humans body [22]. Material hardness of PHA in the range from 3 to 4.5 MPa suggests that it can find applications where elastomeric properties are desired. Polymer materials can release toxic substances into the environment due to their composition or method of manufacturing [23]. Additionally, since PLA can be subject of hydrolysis and released to the medium in form of lactic acid (pKa = 3.86 [20]), it might cause pH change of the medium, and thus influence the behavior of the cells. For PHO the hydrolysis can also occur. However, its products are less acidogenic than for PLA, as these are mostly dimers of (*R*)-3-hydroxyacids (pKa = 4.84 [12]). Parameters such as the manufacturing technique or solvents used in the extraction process can significantly affect the biocompatibility of materials.

The mechanical, physicochemical, and biological properties of the environment affect the structure and morphology of living cells. Cells grown on substrates of different elasticities undergo a series of complex mechanical interactions mediated by focal adhesion and other adhesive structures [24,25]. It is known that some migrating cells (e.g., fibroblasts) react to substrate rigidity by directing movement toward substrates with a higher Young’s modulus value [26]. Cellular structures and functions depend on their microenvironment, which shows a relationship between cellular properties and their immediate surrounding. Sensing the environment’s properties is possible due to interactions of the extracellular matrix (ECM) protein network with transmembrane proteins that can affect the cytoskeleton, enabling the transmission and transformation of mechanical stimuli. Cells are also able to detect and react to the chemical properties of the substrate, such as coverage with proteins, its roughness and chemical composition [27]. Cell movement or motility is a highly dynamic phenomenon that is essential to a variety of biological processes. It is the basis of many physiological events such as the development of a living organism (morphogenesis), wound healing or immunological mechanisms. Still, its deregulation can trigger many pathological processes such as cancer metastasis, chronic inflammation, or neurological diseases. Cell migration is an integral part of cell biology, embryology, immunology, and neuroscience [28]. In case of wound healing, not only the movement of a single cell must be taken into account but the collective motion of many cells. 

The wound healing assay is a standard *in vitro* method of creating a cell-free area on a monolayer of cells, imitating a wound or scratch, which is mostly used to observe collective cell migration in two dimensions [29]. The cells are removed from the indicated area by mechanical, thermal, or chemical damage, creating a gap, into which they migrate, aiming to keep the integrity of the monolayer (or tissue in the in vivo example). The basic information, which is delivered from wound healing assay experiment is the gap closure rate, which is the speed of the collective motion of the cells. The gap closure rate can be measured by simply gathering microscopic images of a scratch fulfillment process starting at the beginning and over the progress of closing this artificial wound [30]. 

Single-cell migration is driven by a different mechanism than tissue regeneration when collective cell movement is taken into account [31,32]. To assess the usability of a polymer for wound dressing analyzing the single-cell motility is not enough. Wound healing assay is an in vivo experiment quite accurately reproducing certain parameters influencing wound healing. It also allows for screening of a range of materials in a fast and repeatable manner. It is often the last stage of experimental research, thanks to which it is possible to switch from an *in vitro* experimental setup into the in vivo studies. For biopolymers examined in this study, namely polyhydroxyoctanoate (PHO) and polylactic acid (PLA), and in order to find a proper application for them in the medical field such as wound dressings, bone implant coatings or surgical sutures, the wound healing assessment was performed. It is no less important to apply numerous physicochemical techniques such as dynamic mechanical analysis (DMA) alongside with contact angle measurements or nanoindentation and combine them with advanced cellular study techniques (confocal microscopy and fluorescent staining protocols) to perform a full material characterization of PHO and PLA in order to explore their biomedical potential. Merging these combinations of physicochemical and biological advanced research techniques may become a benchmark for testing other materials for medical applications.

## 2. Materials and Methods

### 2.1. Materials Preparation

Raw polymeric materials (PHO—laboratory obtained and PLA, MG Chemicals—Ashburton Park, Wheel Forge Way, Trafford Park Manchester, M17 1EH, UK) were dissolved in ethyl acetate (0.5 g of polymer per 10 mL of solvent). Solutions were casted on round bottom glass dishes. Solution (18–21 μL) was poured on glass to get a flat polymer surface of 80–100 μm height. The solvent was evaporated by air drying and the films were left to mature for 14 days. The height of the films covers the range at which the influence of the glass hardness of the substrate on cells is minimized [33]. On the other hand, it enables the observation of cells using a 40× oil immersion objective—in this case the “working distance” parameter is a limitation of maximal substrate height.

### 2.2. Physicochemical and Mechanical Characterization of Polymers

Determination of the hardness and elasticity of PLA samples was performed on round microscope slides covered with a thick (300–500 μm) polymer layer. The thickness of the sample was chosen in such a way that the glass substrate did not affect the measurement result during the measurement process. A polymer microscope slide was attached to the sample holder using the same adhesive for each sample. Measurements were carried out on a Nano Test Vantage device (Micro Materials, Wrexham, UK). The measurements parameters were identical as in previous mechanical studies regarding PHO films [21]. Contact angles were obtained with Drop Shape Analyzer KRUSS DSA100M optical contact angle measuring instrument (Hamburg, Germany, Gmbh) as described earlier [12]. The measurements were made in deionized water. For each concentration, more than three successive measurements were carried out. X-ray pattern was recorded using an X’Pert PRO diffractometer (PANalytical B.V., Enigma Business Park Grovewood Road WR14 1XZ UK) operated at 40 kV and 30 mA by using Ni-filtered Cu Kα radiation. Diffractogram was registered at room temperature in 2θ range of 2–72°_._ The crystallinity degree was calculated after deconvolution of the pattern using Origin Pro 2019 with the equation:

(1) $$\chi_{c,XRD}=\frac{\Sigma_{crystal}}{\Sigma_{crystal}+\Sigma_{amorphous}}$$

Characteristic reflections for mesophase and amorphous halo were found in the literature: 2Ɵ = 16.5°—(110) and (200) lattice plane of the α and α’ crystalline form of PLA; 2Ɵ = 18.8, 23.3 and 28.8°—reflection characteristic for (203), (015) and (216) planes [34]. 

### 2.3. Cell Cultures

Mouse embryonic fibroblasts MEF 3T3 cells were grown in plastic culture flasks under sterile conditions in an incubator (Thermofisher Scientific DH Series, 168 Third Avenue Waltham, MA USA) maintaining constant environment (37 °C, 5% CO_2_). The culture medium used was DMEM (Dulbecco’s Modified Eagle Medium) supplemented with 10% fetal bovine serum (FBS) and 1% antibiotics (penicillin and streptomycin, Sigma-Aldrich^®^ Poznan, Poland). The cell culture was split using a standard passage procedure when the confluence reached about 80%. Cells used in the study were after third, but not beyond ninth passage [35,36].

### 2.4. Cytotoxicity Assessment

A double staining Fluorescein Diacetate/Propidium Iodide (FDA/PI) test was used for the cytotoxicity assessment in order to distinguish between dead and living cells [37]. After staining, the cells were counted and the level of viability was assessed. Briefly, cells were detached from the substrate with trypsin, cell suspensions were centrifuged and excess medium removed so that pellet remained in the Eppendorf tube. The cell pellet was resuspended in 100 μL of staining solution and then placed on microscope slide and imaged under the microscope. 

### 2.5. Actin Cytoskeleton Staining

To stain actin fibers, cells were fixed using cross-linking method with application of formaldehyde as bonding agent, then a dye solution containing rhodamine-labeled phalloidin (Sigma Aldrich^®^, Poznan, Poland) was applied. After that, light shielded samples were left in the refrigerator overnight (5 °C) until the imaging could be performed. The imaging was performed using Plan-Apochromat 40×/1.4 Oil DIC M27 objective (Carl-Zeiss-Promenade 10, 07745 Jena, Germany) and the wavelength of the excitation laser was 540 nm and detection range was set to 500–643 nm. 

### 2.6. Quantitative Cytoskeleton Analysis

The cytoskeleton analysis was conducted using ImageJ (Fiji) ver. 1.51h software (9000 Rockville Pike, Bethesda, Maryland, USA). The subject of analysis was the actin cytoskeleton. Image analysis, and applied algorithm were performed as described in earlier work [21]. To quantify differences in cell cytoskeleton on different substrates, the density of actin fibers in each cell region was analyzed. 

### 2.7. Migration Analysis

Migration analysis based on microscopic observations performed in fluorescent and bright field modes on Zeiss Axio Observer Z.1 fluorescent microscope (Carl-Zeiss-Promenade 10, 07745 Jena, Germany). Cell tracking was executed using Cell Tracker v 1.0 Software (9000 Rockville Pike, Bethesda, Maryland, USA). The cell tracking protocol, intravital staining of the cell nucleus were performed as described in earlier studies concerning PHO films [21] using NucBlue (Thermo Fisher Scientific, 168 Third Avenue Waltham, MA USA) reagent based on Hoechst 33342 reagent. Migration analysis experiment lasted for 24 h during which a series of microscopic images were recorded with time resolution of 5 min. 

### 2.8. Wound Model

To thoroughly investigate the influence of the substrate on the tissue healing process, additional studies were carried out using a standard wound model [30,38]. To prevent the PLA films from damage, we modified the mechanical scratch method by using a thin parafilm strip wrapped tightly around the sharp end of the pipette tip. This way a soft, thin shield on top of a pipette tip was created that was compact enough, to create a repeatable 400–500 μm gap in a monolayer of cells, but at the same time accurately gentle, to avoid tearing of the PLA film. Cell growth was performed inside glass bottomed dishes until the confluency reached 100% (monolayer), microscopic observations were performed using the same experimental setup as for migration analysis. Preservation of specified and constant conditions (i.e., time of cell growth into monolayer, same method for creating a gap, the same angle while performing a scratch, etc.) during each wound healing assay allow obtaining comparable and repeatable results. Wound healing rate can be calculated by measurement of the width of the gap created for the experiment in a function of time. The two main values that can be obtained from that experiment are:

(2) $$t_{1/2\;gap}=\frac{Initial\;Gap\;Area}{2\times \left|slope\right|}$$

(3) $$v_{migration\;}=\frac{\left|slope\right|}{2\;\times \;l}$$

$v_{migration\;}$ is the average velocity of cells movement into the gap [µm/hour], whereas $t_{1/2\;gap}$ (cell sheet migration rate) is the parameter that can only be used as a comparator for experiments, in which wound width is the same [39].

Experimental data were analyzed using diCELLa (diCella, Poland) Scratch Assay software. To increase the statistical significance, for all examined materials separate regions (5–7) of scratch were analyzed on each culturing dish. In parallel, experiments were carried out in reference vessels where the cells were fixed, nucleus and actin fibers were stained at selected time intervals after creating the gap [21].

## 3. Results and Discussion

### 3.1. Preliminary Materials Assessment 

To assess the suitability of given materials in biomedical applications, it is essential to precisely determine their impact on living organisms. The first step is to perform assessment of their cytotoxicity followed by studies of migration behavior of cells, cultured on them and then to correlate of the latest with material properties of the tested specimens. Since both PLA and PHA can potentially be used as a wound dressing material or absorbable surgical sutures, it was decided to use mouse embryonic fibroblasts (MEFs) as a model cell line for cytotoxicity tests because fibroblasts play an important role in wound healing processes, and are common in the body [40,41].

**Figure 1:**
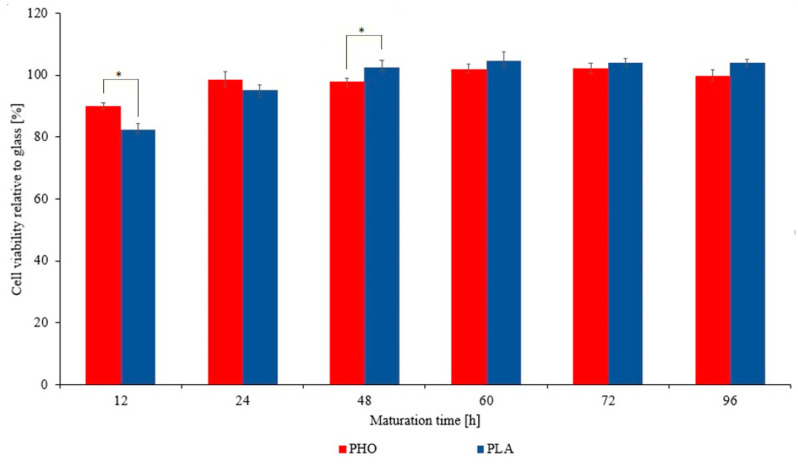
Comparison of PHO and PLA cytotoxic activity relative to glass. Data for glass and PHO was taken from earlier study [21]. The horizontal axis shows the time elapsed since pouring the polymer. After 24 h the atrophy of the influence of the evaporating solvent on cell viability can be seen. Results statistical significance: * *p* < 0.05

The cytotoxicity results obtained for PLA were compared directly with the PHO and a standard glass bottomed dishes, which were used for cell breeding and presented as a reference. Cells cultivated on PLA showed no cytotoxic effect as it was also the case for pure PHO polymer thin films [21] (Figure 1). Thus, the high cell viability index, similar to glass bottomed dish, indicates that both polymers can be used in medical products and come into direct contact with living cells. 

Migration is an important process associated with numerous physiological phenomena such as wound healing and tissue regeneration [42,43,44,45]. Therefore, it was necessary to evaluate this process on the surface of polyesters to assess their usefulness for the production of biomedical devices such as wound dressings or tissue scaffolds. The performed studies revealed that PLA and PHO are biocompatible materials. Because the material’s Young’s Modulus value can affect the cell behavior [46], and since this value for PLA is by 3 orders of magnitude higher than for PHO, one can expect that migration strategies of MEF 3T3 cells will differ on both materials. In the literature, numerous studies show that migration strategy can vary depending on type of material [47,48,49,50], pore sizes [51,52], substrates microarchitecture [53] or substrates manufacturing method [54]. Cell’s microenvironment pH is also a factor which may alter the cell migration and thus wound closure dynamics [55]. 

**Figure 2:**
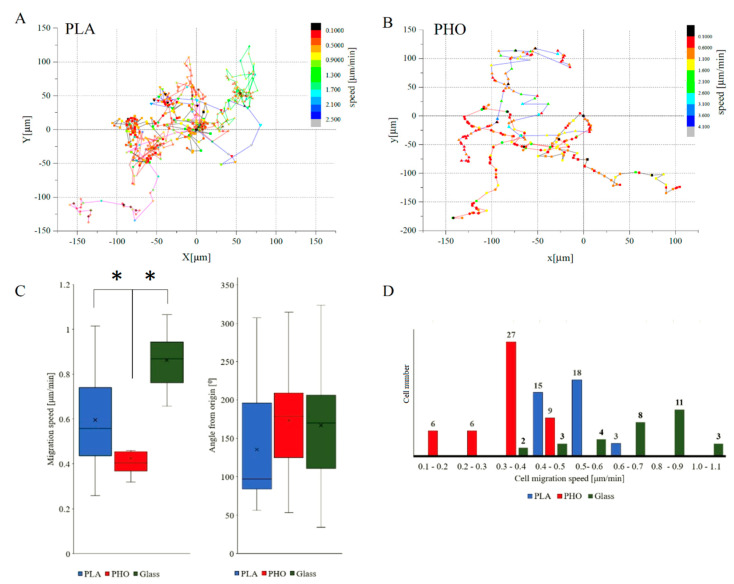
Migration analysis of MEF 3T3 cells grown on PLA and PHO. Selected trajectories of cell movement (**A**-PLA, **B**-PHO) along with instantaneous speeds at each time point, comparison of average velocities and migration angles (**C**) as well as histograms showing the distribution of migration velocities (**D**), numbers over the bars represent number of cells migrating at certain speed range. Data for both biopolymers was compared with glass [21] as reference material. Results statistical significance: * *p* < 0.05

Cells migrating on PHO biopolymer exhibited similar parameters to those grown on PLA substrate (48 cells for PHO tracked and analyzed versus 36 for PLA). Migration speed was visibly lower while compared to glass by 30% for PLA and 45% for PHO (Figure 2C). High spread of values of “angle from origin” parameter proves lack of directional migration (Figure 2, Panel C). This effect is also visible in figures presenting tracks of individual migrating cells (Figure 2, Panel A and B). Migration velocity distribution for each experimental group has been presented on histograms (Figure 2D) to visualize whether there was a dominant group of cells at a given migration speed on the examined substrate or if the distribution was homogeneous. For cells migrating on PHO material, the dominant group (56%) was constituted from cells migrating with velocities between 0.3 μm/min and 0.4 μm/min. In the case of PLA, most cells (92%) migrate with speeds in the range of 0.3 μm/min and 0.5 μm/min. These observations can be related directly to materials physicochemical properties. First of all, on a less crystalline PLA (X_C,DSC_ = 4%), single-cell movement was noticeably slower compared to semi-crystalline PHO (X_C,DSC_ = 37%). This correlation was also visible when the water wettability of both materials was compared against the migration speed. We measured that contact angles for PLA and PHO films were 68° ± 2° and 100° ± 6°, respectively. According to the literature, materials with higher hydrophilicity are characterized by a better capacity for protein adsorption, improved cell adhesion as well as proliferation [56].

### 3.2. Wound Healing Model

After conducting migration studies to verify how the material properties affect the cell movement itself, the next stage was to perform a wound healing assay on our tested materials. This type of experiment was selected because it shows the direct impact of the material on cells in external conditions which simulate a real wound very thoroughly. While using a wound healing model, not only can the migration processes be observed, but also the dynamics of wound closure can be analyzed at any stage of the experiment. It was possible to quantify the entire process thanks to the application of specialized dedicated to this kind of analysis diCELLa Scratch Assay software.

**Figure 3:**
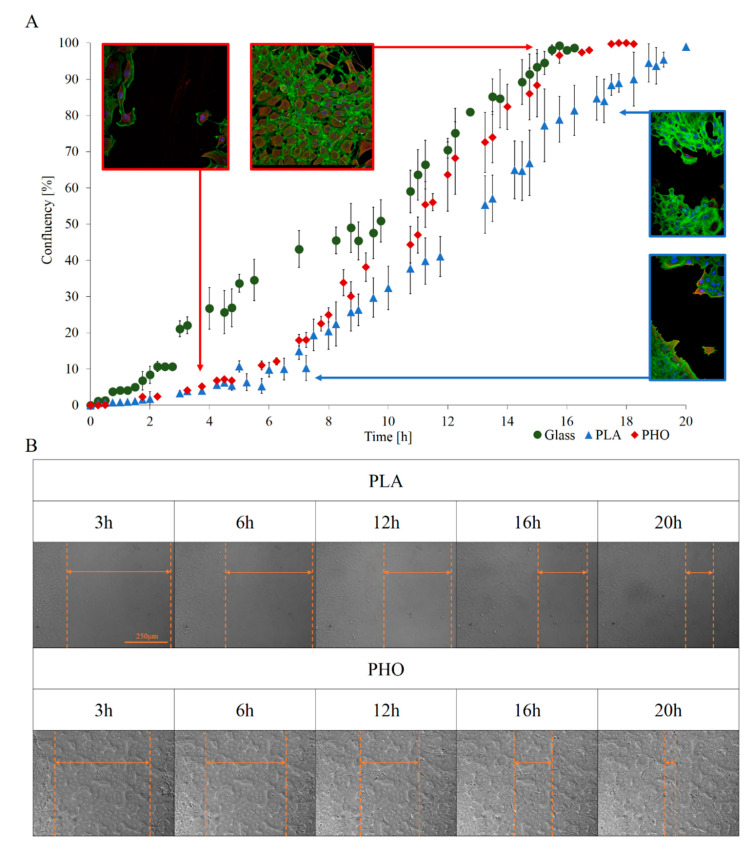
Wound healing assay results. Quantitative analysis allowed to show and analyze the dynamics of wound healing (**A**), and thanks to the use of high-resolution microscopic imaging wound healing and migration of cell groups to its center can be observed at selected timepoints (**B**). Cells for selected timepoints were fixed and stained with solution containing rhodamine-labeled phalloidin (Sigma Aldrich) to dye the actin fibers. DAPI (Thermo Fisher Scientific) was used for nucleus staining.

The first observation was that the progress of wound closure is much slower for PLA than for PHO. MEF 3T3 on PHO presented 100% relative confluency after approximately 16 h whereas on PLA the process took about 22 h, on glass the process took 16h. The other tendency related to the beginning of the healing process was the slower, nonlinear confluency progress in case of both biomaterials, which becomes higher after about 3 h. Subsequently, the process becomes linear in case on both biopolymers. In case of glass substrate, the closure process was linear throughout the entire experiment. It seems that cells grown on PLA and PHO (Figure 3A) start the healing process slowly, and the process slows down when the confluency reaches about 90%. The global dynamic of wound closure for PHO is higher than for PLA, and after 16 h the wound was fully closed such as in case of glass. These observations are non-consistent with single-cell migration analysis where cell movement was slower by about 25% on PHO against PLA substrate and 45% against glass. This is not contradictory, since wound healing process is a collective phenomenon where novel cellular interactions which are not present in the case of single, separated cells, occur. Another reason for this may be the fact that cells can take nutrients not only from the culturing medium but also from the material itself. The release of monomers from biomaterials has been confirmed in the previous works [12]. Higher migration speed on PLA may be affected by higher YM value. It has been shown in earlier works that material stiffness can impact the cells migration speed [46]. In the case of collective migration, which is exemplified by the wound healing process, the tendency is the opposite. The cells on the PHO substrate eventually overgrow the wound faster than on PLA. This can be influenced by the composition of the substrate, supporting their proliferation in this case. An additional factor may be the pKa values of both materials that affect the pH of the cell microenvironment.

### 3.3. Quantitative Cytoskeleton Analysis

**Figure 4:**
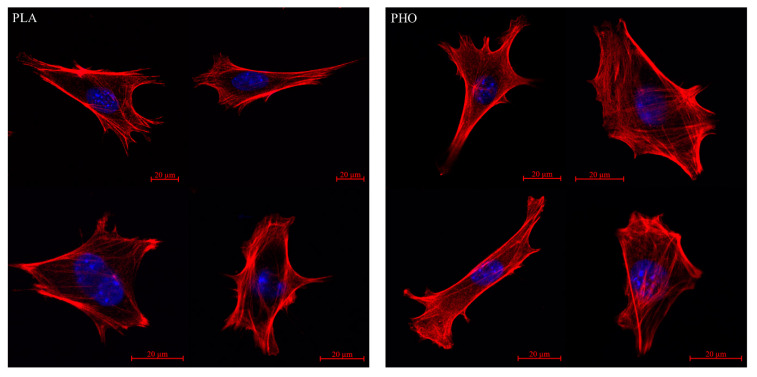
Exemplary microscopic images showing the structure of actin fibers in cells cultured on PLA (left) and PHO (right) substrates.

**Figure 5:**
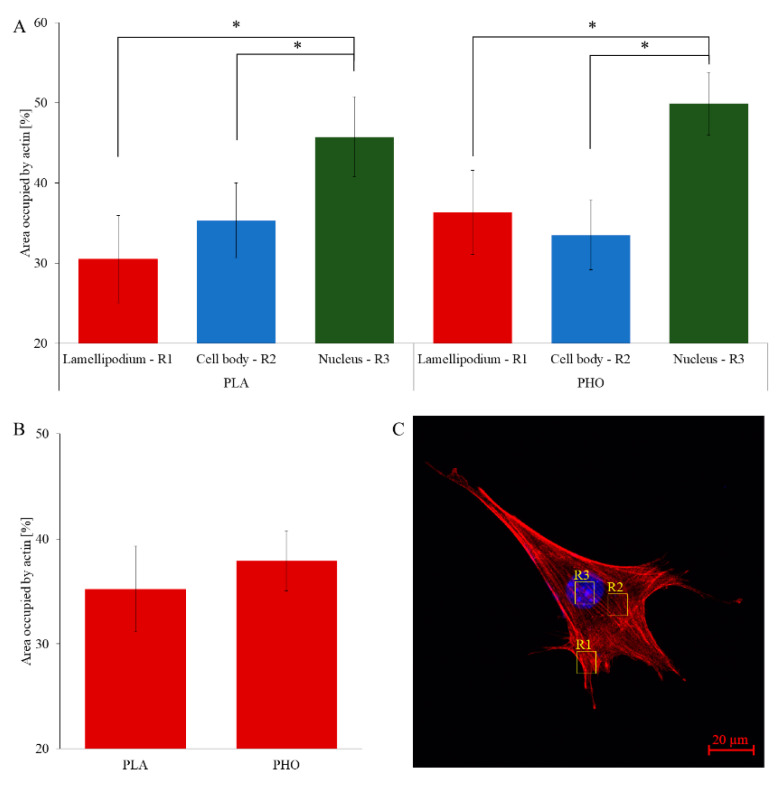
Comparison of area occupied by actin divided into individual cell regions and substrates (**A**), compared with the area occupied in the whole cell (**B**). Exemplary regions taken into consideration during the analyses are depicted in panel (**C**): R1—lamellipodium, R2—nucleus, R3—cell body. In total 35 cells for PLA and 40 cells for PHO have been examined. Data for PHO sourced from [21]. Results statistical significance: * *p* < 0.05

High-resolution microscopy imaging allowed us to analyse the cell conditions in real time. Conducting parallel experiments to the wound healing assay with cell fixing and fluorescence staining enabled visualizing the exact shape of the actin cytoskeleton and nucleus at selected time points. Images obtained during the test, did not reveal any significant differences between cells grown on PLA and PHO. On both materials, cells were well spread and presented a morphologically correct shape (Figure 4). Both PLA and PHO did not affect the interaction of the cells with the substrate. The microscopic observations confirm excellent biocompatibility of both materials for MEF 3T3 cells. During the data analysis, the attention was focused on the arrangement of the fibers in the cell body and their distribution. The area occupied by actin fibers was calculated and compared between cells grown on different substrates. The obtained data indicate that in both “regional” (Figure 5A) and global (Figure 5B) approaches, cells cultured on both types of biopolymer exhibit thin but densely spaced actin bundles. Actin filaments in cells grown on both PLA and PHO often create an intricate network with many bifurcations. In the “regional” analysis, the actin cytoskeleton for cells grown on PHO takes up slightly more space than analogically on PLA. Still, the differences are within the limits of the standard deviation for the results group, so they cannot be treated as statistically significant (Figure 4). In the case of whole-cell analysis, this differentiation disappears. It is a known fact that studied cytoskeletal structures, as well as the cells themselves, are dynamic objects and their behavior and morphology depend on several different factors that are not included in the study [57,58,59,60]. However, during the experiments, efforts were made to minimize the influence of external factors through identical culturing conditions, passage and staining protocols, or microscopic observation parameters itself.

## 4. Conclusions

The material properties of biopolymers play a significant role in the processes associated with cell adhesion and migration. Biological research shows that both PHO and PLA are biocompatible materials. The obtained parameters throughout the study were gathered in Table 1. The average migration velocity of single MEF 3T3 cells is lower by 25% in case of PHO when compared to PLA and 45% while compared with glass, at the same time, materials do not interrupt the direction or strategy of cell movement. The differences between the behavior of cells on PHO and PLA become much more pronounced if the process of wound healing is being analyzed when the aggregate movement of the cells clusters and tissue formation is observed but not the movement of single cells. Under conditions established during the tests, a 400–500 μm wide wound in the case of PLA closed after almost 20 h. In the case of PHO substrate, the dynamics of the healing process were similar to glass and distinctively different to PLA. The growth speed was higher, and the gap becomes overgrown in about 17 h. Both PHO and PLA have great potential in the biomedical industry. The research shows that both bio polyesters are promising candidates for preparation of wound dressing materials. PHO can be applied “as is” thanks to its elastomeric properties, whereas PLA needs further processing due to its brittleness. Nevertheless, both materials promote cell adhesion, growth, migration leading to complete wound closure, when tested *in vitro*. Our findings about the performance of cells on these biopolymers in the future can be directly applied to wound healing. This process takes place in four stages: haemostasis, inflammation, proliferation, and remodeling [61]. The application of PLA or PHO without any modifications will surely contribute to the support of controlled wound closure by facilitating the cell migration into the wound. Moreover, these biopolymers can be easily modified with different drugs, e.g., anti-inflammatory, and this in turn can contribute to a programmable inflammatory process (e.g., by slowing it down or stopping it in a controlled manner). Nevertheless, these demands require further research, and the first step may be an explant *ex vivo* model before clinical applications.

## Figures and Tables

**Table 1:** Summary of obtained results for MEF 3T3 cells studied on two biopolymers.

	Parameter	Cytotoxicity after 96 h of Maturation *	Single-Cell Migration Speed [um/min]	Wound Closure Time [h]	Area Occupied by Actin			
Material					Lamellipodium	Cell Body	Nucleus	Total
PLA		101.3% ± 1.8	0.63 ± 0.22	20.5	30.51 ± 4.93	34.73 ± 4.15	45.97 ± 4.42	34.70 ± 4.35
PHO		99.6% ± 1.2	0.43 ± 0.12	16.4	35.11 ± 5.14	31.68 ± 4.26	49.66 ± 3.91	38.32 ± 3.12
Glass		100%	0.82 ± 0.21	16.2	44.75 ± 4.71	60.35 ± 3.89	51.98 ± 3.32	49.66 ± 3.09

* relative to glass; data obtained in this study: PLA—all parameters, PHO and glass—wound closure time; other parameters for glass and PHO sourced from [21].
